# Supplementary material for: An Integrated Mycobacterium tuberculosis Infection Session: Utilizing an Online Collaborative Platform in a Synchronous Classroom Setting
Source: MedEdPORTAL. 2021 Apr 14;17:11143. doi: 10.15766/mep_2374-8265.11143 (PMC8056773; doi:10.15766/mep_2374-8265.11143)
Supplement: Supplementary file 1 — Student Mycobacterial Defense Mechanisms Spreadsheet.xlsxCloud-Based Learning Detailed Description.docxPretest and Posttest MCQs.docxMycobacterium tuberculosis Defense Mechanisms.pptxInstructor Mycobacterial Defense Mechanisms Spreadsheet.xlsxFeedback Guidelines for Cloud-Based Learning.docx [file mep_2374-8265.11143-s001.zip › B. Cloud-based Learning Detailed Description.docx]

**Cloud-based Learning Detailed Description**

**(Numbering corresponds to ESR Figure)**

1. Students take a 10-minute formative pretest consisting of 8 multiple choice questions to determine students' baseline levels of knowledge (Appendix C). Students answer these questions before class begins or at the beginning of the session in the learning management system (i.e Moodle). As these questions are used to establish a baseline knowledge before the session, the students do not get feedback to the answer choices at this time.
2. A 10-minute didactic lecture is given to review concepts of mucosal immunity in the respiratory tract (Appendix D, slides 1-15).
3. During this 35-minute time period, students use a cloud-based spreadsheet (Appendix A) containing several open-ended questions related to *M. tuberculosis.* When the collaborative spreadsheet is shared with students it is important that students have editing privileges for the document. Four facilitators (faculty and student assistants) who had covered the subject the previous year provide real-time feedback from an instructor version of the spreadsheet with all correct answers provided as a guide (Appendix E).
   1. Student Groups: Students work in groups with their classmates, as described in the participants section of the methods, to retrieve, analyze, and synthesize the information from credible online sources such as MedlinePlus® or UpToDate®, or journal articles, and answer their assigned question in the class spreadsheet (Appendix A). As students discuss answers and questions with their group members, they give and receive peer-peer feedback on their sources and answers.
   2. Instant Faculty Feedback: Prior to the session, all facilitators are sent an email with links for the spreadsheets (Appendices A and E), as well as a training document containing examples of how to provide guided-instant feedback (Appendix F). It is important for faculty to have both the student version and the instructor version of the spreadsheet open simultaneously on their own computers while providing this feedback. Some instructors provide feedback while present in class (those who are in charge of the session) while others find it more convenient to work remotely (students assistants or other faculty). In the student version of the spreadsheet (Appendix A), there are sections called ‘Feedback to Group’ in which faculty can provide direct real-time feedback to students using appendix E. Appendix E not only contains the correct answers, but also contains additional questions in the ‘Feedback to Group’ section that faculty can ask the students to promote deeper understanding of the material. The answers to these additional questions are provided in Appendix E to help the instructors evaluate the accuracy of students' answers. A 10-minute break is then provided.
4. During the next 35-minute session, faculty integrate microbiology and immunology concepts about *M. tuberculosis* (Appendix D, slides 17-38). They review and provide class-wide feedback on the material covered in the cloud-based portion (section 3 above) by comparing the slideshow (Appendix D) to the student-created study-guide (Appendix A). Next, students answer multiple choice questions using polling software. Our students use school-supplied iClickers©, although any polling software such as Poll Everywhere, Turning Point, Socrative, etc., could be used to gauge student learning from the cloud-based portion (Appendix D, slides 24-25, 35-36, 40, and 43).
5. A 10-minute formative posttest assesses learning and retention of the concepts covered during the session (Appendix C). Each question has the rationale for the correct answer choice providing valuable feedback to students. If presenters need more time to complete the session, this formative posttest could be assigned to be done after class, thus allowing an extra 10-minutes.
6. After the session, faculty review the student-completed version of Appendix A for accuracy and completion, and then post it online in the learning management system for all students to use as a study guide.
