# Supplementary material for: An Integrated Mycobacterium tuberculosis Infection Session: Utilizing an Online Collaborative Platform in a Synchronous Classroom Setting
Source: MedEdPORTAL. 2021 Apr 14;17:11143. doi: 10.15766/mep_2374-8265.11143 (PMC8056773; doi:10.15766/mep_2374-8265.11143)
Supplement: Supplementary file 1 — Student Mycobacterial Defense Mechanisms Spreadsheet.xlsxCloud-Based Learning Detailed Description.docxPretest and Posttest MCQs.docxMycobacterium tuberculosis Defense Mechanisms.pptxInstructor Mycobacterial Defense Mechanisms Spreadsheet.xlsxFeedback Guidelines for Cloud-Based Learning.docx [file mep_2374-8265.11143-s001.zip › C. Pretest and Posttest MCQs.docx]

**Pre- and Posttest Questions for the Integrated Respiratory Session**

**Note:** Questions are provided using a learning management system. For the pretest, do not provide the answers. **For the posttest, please provide the correct answers and rationale.**

**1. Which of the following population groups is the most at risk for TB disease?**

A. Adults over age 65

B. Children aged 5-12

C. Health care workers

D. Prostitutes

E. Veterinarians

Correct Answer: A, adults over age 65 fall into the immunocompromised category.

**2. Which of the following distinguishes TB disease from LTBI (Latent TB Infection)?**

A. Bacteria can be transmitted to others in TB disease (not for LTBI).

B. IGRA test is positive in TB disease (negative for LTBI).

C. PPD test is positive in TB disease (negative for LTBI).

D. Resistance is common for TB disease (not for LTBI).

E. Treatment should be considered for TB disease (not for LTBI).

Correct Answer: A, bacteria is transmitted to others during TB disease, but not during a latent infection.

**3. Which of the following antigens is diagnostic and is also used to differentiate patients infected with TB from those who have received the BCG vaccine?**

A. Mycolic acid

B. TDM

C. LAM

D. ESAT-6

E. Wax D

Correct Answer: D, ESAT-6, CFP-10, TB7.7 are the specific antigens that are found in TB (use for this assay) but not BCG.

**4. What would be expected in terms of the humoral immune response upon nasal vaccination using FluMist?**

A. Lactating mothers will provide antigen-specific natural IgA in breast milk.

B. Secretory IgM will be synthesized in the lamina propria of all mucosae.

C. It does not enter the bloodstream, but instead remains in the mucosa and differentiates into an effector B cell.

D. B-cell producing IgA will be found only in the respiratory tract where the Ag was first taken up.

E. Monomeric IgA is secreted into the lamina propria.

Correct Answer: A, nasal vaccination activates the immune response from the mucosa, which leads to formation of B cells which migrate systemically towards the mammary glands. In humans, immunization by different mucosal routes shows that the strongest response takes place at the directly vaccine-exposed mucosa and the second-best responses at the closest mucosae or interconnected inductive-expression mucosal systems (GI, mammary). Nasal mucosal immunization not only stimulates an immune response in the respiratory tract, but also can give rise to a strong genital-vaginal mucosal immune response.

**5. A 15-year-old male from South America presented with poor appetite, diarrhea, progressive weight loss (2 months), enlarged mesenteric and mediastinal lymph nodes. Blood exam revealed slightly increased neutrophils, IgA and IgG levels. Biopsy of lymph nodes showed marked proliferation of histiocytes, but not granuloma and giant cells formation. PPD was negative despite being vaccinated with BCG, and a positive acid-fast intracellular pathogen on a sputum smear. Which of the following diseases is most likely the diagnosis?**

1. X-linked hypogammaglobulinemia
2. CD40 deficiency
3. IgA deficiency
4. Deficiency of IFN-gamma receptor
5. C1q deficiency

Correct answer: D, this is a case of TB in which the patient is unable to form granulomas because macrophages do not have the IFN-gamma receptor. Meaning that macrophages cannot be adequately activated to contain the pathogen.

**6. A relatively specific clinical phenotype characterized by increased susceptibility to TB, suggests most likely a problem in which of the following?**

1. The TH1 cell component
2. The TH2 cell component
3. The NK cell component
4. The complement component
5. The B cells component

Correct answer: A, CD4+ T-cells, specifically TH1, are important in producing IFN-gamma to activate macrophages. In tuberculosis, CD4 T-cells help macrophages to contain the pathogen forming a granuloma.

**7. A 45-year-old male presented with a two-month history of pneumonia caused by PCP and several lesions characteristic of Kaposi sarcoma. His history showed that he was diagnosed with HIV and TB, 2 and 20 years respectively, and his vaccines are up to date. Which of the following statements is most likely correct for this patient?**

1. PPD positive, normal titers of IgG anti Tdap
2. PPD negative, low levels of IgG anti Tdap
3. PPD positive, low levels of IgG anti Tdap
4. PPD positive, normal levels of IgG anti TB
5. PPD negative, normal levels of IgG anti TB

Correct answer: B, this case describes an AIDS stage HIV patient, in which there would be a low CD4 count (<200). Making this individual susceptible to PCP, Kaposi Sarcoma, CMV etc. During Low CD4 counts, TB cannot be contained in the granulomas and it spreads everywhere (extrapulmonary TB). PPD is a delayed hypersensitivity reaction (Type IV) that requires CD4 T-cells. An HIV patient in AIDS stage will have low CD4 T-cells, resulting in a false negative PPD reaction.

**8. A 52-year-old male presents to his physician with a persistent cough, bloody sputum, low-grade fever, chest pain, extreme fatigue, and night sweats. The causative agent was confirmed with a positive acid-fast stain of a sputum sample. Which of the following test results would be diagnostic and specific for this patient’s disease?**

A. Chest X-ray

B. Gram stain

C. Medical history

D. PPD reaction

E. QuantiFERON test

Correct Answer: E, QuantiFERON test is specific in detecting individuals who have been infected by tuberculosis. PPD reaction detects those that have been immunized with BCG vaccine, or have tuberculosis.
