# Supplementary material for: An Integrated Mycobacterium tuberculosis Infection Session: Utilizing an Online Collaborative Platform in a Synchronous Classroom Setting
Source: MedEdPORTAL. 2021 Apr 14;17:11143. doi: 10.15766/mep_2374-8265.11143 (PMC8056773; doi:10.15766/mep_2374-8265.11143)
Supplement: Supplementary file 1 — Student Mycobacterial Defense Mechanisms Spreadsheet.xlsxCloud-Based Learning Detailed Description.docxPretest and Posttest MCQs.docxMycobacterium tuberculosis Defense Mechanisms.pptxInstructor Mycobacterial Defense Mechanisms Spreadsheet.xlsxFeedback Guidelines for Cloud-Based Learning.docx [file mep_2374-8265.11143-s001.zip › F. Feedback Guidelines for Cloud-based Learning.docx]

**Feedback Guidelines for CL**

- When giving feedback state whether the student’s answer was correct, incorrect, or partially correct.
  - If incorrect, point out why what they entered was incorrect.
  - If incorrect, point out a piece of reasoning they need to use in order to get to the correct answer.
- Examples of phrases to input when students’ answers are completely correct
  - Correct! Please move on to the questions in row X
  - Great job! You have finished this section, please move on to the questions in row X.
- Examples of phrases to input when students’ answers are partially correct
  - Some of that is true.. But what about ____?
  - A good thought, however…
  - How would this affect ___.
  - What kind of effect would _____ cause?
- Examples of phrases to input when students’ answers are incorrect
  - That is incorrect because ____.
  - Think more along the lines of ____.
- Phrases will be in a completed spreadsheet for you to copy and paste into the students’ document in order to save time.
