# Supplementary material for: An Integrated Mycobacterium tuberculosis Infection Session: Utilizing an Online Collaborative Platform in a Synchronous Classroom Setting
Source: MedEdPORTAL. 2021 Apr 14;17:11143. doi: 10.15766/mep_2374-8265.11143 (PMC8056773; doi:10.15766/mep_2374-8265.11143)
Supplement: Supplementary file 1 — Student Mycobacterial Defense Mechanisms Spreadsheet.xlsxCloud-Based Learning Detailed Description.docxPretest and Posttest MCQs.docxMycobacterium tuberculosis Defense Mechanisms.pptxInstructor Mycobacterial Defense Mechanisms Spreadsheet.xlsxFeedback Guidelines for Cloud-Based Learning.docx [file mep_2374-8265.11143-s001.zip › _Educational_Summary_Report_11143.pdf]

MedEdPORTAL® The AAMC Journal of Teaching and Learning Resources

Original Publication Open Access

An Integrated *Mycobacterium tuberculosis* Infection Session: Utilizing an Online Collaborative Platform in a Synchronous Classroom Setting

Trent D. Jackman, Anne M. Dersch, MD, Tracey A. H. Taylor, PhD, Claudio Cortes, DVM, PhD*

*Corresponding author: cortes@oakland.edu

Abstract

Introduction: Tuberculosis (TB) remains a major public health concern worldwide. It is important to provide high-quality instructional sessions to students about the pathogenesis and risk factors of TB, as medical students are likely to encounter TB infections in clinical practice. **Methods:** We describe an interactive instructional session integrating immunology and microbiology concepts of *Mycobacterium tuberculosis* infection that was presented to first-year medical students in their respiratory organ systems course. The session included a pretest primer followed by a brief review of mucosal immunity with an emphasis on the respiratory system. Using an online collaborative application, learners created a study guide on a shared spreadsheet while faculty provided real-time feedback. Following the cloud-based portion, faculty presented interactive lectures using student-created content. The session concluded with a formative posttest. We evaluated the session with responses to an optional student survey. **Results:** One hundred fourteen students (37% male and 63% female) completed the survey across 4 years from 2016 to 2019. The session received high student satisfaction ratings across five questions, with 83% of students indicating they were slightly satisfied to strongly satisfied. The students had an absolute increase in their scores of 31% on the posttest as compared to the pretest mean ($p < .001$). **Discussion:** We developed an interactive TB instructional session that integrates disciplines, contains real-time instructor feedback, and promotes teamwork in a large class setting. The session allows medical students to learn content and create their own study guide using online collaboration technology.

Keywords
Microbiology, Immunology, Synchronous Teaching, Cloud Learning, *Mycobacterium tuberculosis,* Mucosa Immunity, Interdisciplinary Medicine, Allergy and Immunology, Pulmonary Medicine, Virtual Learning

Educational Objectives

By the end of this activity, learners will be able to:

1. Describe the immunological and microbiological mechanisms and clinical aspects of tuberculosis (TB).
2. Describe prevention, control, and diagnosis for TB infections.
3. Discuss global, national, and local epidemiology of TB (incidence, transmission, and high-risk groups).
4. Discuss microbiological principles for therapy and drug resistance in TB.
5. Develop a student study tool on a shared document using online collaboration software in a large class setting.

Citation:
Jackman TD, Dersch AM, Taylor TAH, Cortes C. An integrated *Mycobacterium tuberculosis* infection session: utilizing an online collaborative platform in a synchronous classroom setting. *MedEdPORTAL.* 2021;17:11143.
https://doi.org/10.15766/mep_2374-8265.11143

Introduction

In 1989, the Centers for Disease Control and Prevention (CDC) established a goal to eliminate tuberculosis (TB) in the United States by rapidly identifying and treating recognized cases by 2010.[1] Unfortunately, the CDC reported more than 9,000 active TB cases in 2018[2] and estimated that approximately 13 million people in the United States may have latent TB infections.[3] While rates are low in the United States, TB is one of the top 10 causes of death globally and is the leading cause of death from a single infectious agent, killing 1.5 million annually.[4] Although TB is highly curable, infection rates remain high worldwide.[5] Drug-resistant strains such as rifampin-resistant TB are increasing, with roughly half a million new cases each year, but only a minority of patients are diagnosed and treated.[4] The *Lancet* Commission on TB identified poor quality of health care as one of the reasons that TB continues to plague many parts of the world.[6] In addition,

Copyright © 2021 Jackman et al. This is an open-access publication distributed under the terms of the Creative Commons Attribution license.

a recent study showed that medical student knowledge of TB is lacking,[7] even as more students are participating in global health.[8,9] This discrepancy emphasizes the need to provide high-quality instructional sessions to medical professionals about TB's bacteria, pathogenesis, and risk factors.

Cloud-based technology has created a paradigm shift in how students use computers in an educational setting. Multiple users can edit and view the same document or spreadsheet on their personal computers simultaneously, and all users can view the updates in real time, even if users are not in the same location. This new technology fosters innovative collaboration and team learning.[10] In undergraduate classrooms, students have used cloud technology (Google Drive) to fill out physics lab reports and submit them to their professor, who tracks the contribution of each group member using the document's revision history.[11] Online technology has been used to facilitate semester-long student collaboration with prompt instructor feedback for an engineering design project.[12] Students who used online collaborative tools to take notes collaboratively in groups improved their grades and classroom performance.[13] These self-directed or directed-independent learning tools allow students to learn at their own pace, spend less time in the classroom, spend more time preparing prior to lecture, and interact with peers digitally.[14]

A recent search of *MedEdPORTAL* for resources teaching foundational immunology and microbiology concepts of *Mycobacterium tuberculosis* yielded limited results. One resource discusses seizures secondary to isoniazid overdose, a common drug used in the treatment of *M tuberculosis.*[15] Another publication is a software-based case presentation where students interpret diagnostic tests that they choose to order and come to one of two diagnoses, pulmonary TB or pneumocystis pneumonia.[16] Still another is a course designed to introduce students to topics in global health, with one of the sessions focusing on HIV and TB.[17] The most recent publication concentrates on interprofessional education and guiding collaborative health care teams through a public health outbreak of TB.[18] To date, no publications focus on the pathogenesis and immunologic mechanisms that control TB, which is an important part of the preclinical curriculum during medical school as resistant TB increases worldwide.[4]

Our goal was to develop an interactive instructional session on the topic of TB that allowed undergraduate medical students to integrate immunology and microbiology while practicing directed-independent learning using technology. This session utilized constructivist principles requiring little student advance preparation, provided formative assessments, and promoted instant formative feedback from both professors and peers. In addition, the session employed technological pedagogical content knowledge (TPACK) and generative learning frameworks to promote a good learning environment,[19,20] resulting in participants creating their own study guide.

Methods

Participants

Participants were first-year undergraduate medical students at the Oakland University William Beaumont School of Medicine (OUWB) enrolled in the winter semester respiratory organ systems course during 2016-2019. Medical students were divided into groups/teams of four to six and were familiar with working in these small groups from previous experience with team-based learning.

Description of Advance Preparation Resources

Prior to participation in the session, medical students received an email notification from participating faculty providing brief information about the upcoming instruction (the link to access the spreadsheet; a basic outline/description of the instructional session; a statement explaining that the session was not mandatory, that participation in the study was optional and would not impact their grade, and how to consent; and contact information for study personnel). Each student at OUWB was given a computer laptop with institutional access through Google Drive (alternatively, any other online collaboration software such as Microsoft OneDrive would work) to the spreadsheet created for the session (Appendix A), as well as access to reliable third-party web information tools available through the medical library (UpToDate, DynaMed Plus). If participants were unable to locate the information needed, they were instructed to find it by accessing web information search engines (Wikipedia, Google, etc.). This information, including recommended readings in textbooks, was also posted in the learning management system for easy access by all students. Two basic science faculty members were responsible for presenting the integrated session on the topic of respiratory immunology and microbiology, with an emphasis on *M tuberculosis*.

Classroom Setting

At our institution, two main classroom types accommodated the entire class of first-year students. Both were used in different years of this session's implementation. One was an auditorium with a single screen at the front of the room; the other was a classroom designed for small-group discussion, with 16 tables and four sets of dual screens. We preferred the classroom with

Copyright © 2021 Jackman et al. This is an open-access publication distributed under the terms of the Creative Commons Attribution license.

the dual screens so that the student-created spreadsheet and faculty slides were both presented simultaneously during the integrated lecture.

Session

Organized on the student schedule as two sequential 50-minute integrated sessions of microbiology and immunology, the session was part of the standard curriculum for the first-year undergraduate medical student respiratory organ system course. Prior to the implementation described here, the sessions had not been integrated and were two separate traditional 50-minute didactic lectures. The major topics of the joint session were mucosal immunity of the respiratory tract and *M tuberculosis* infections, including clinical presentation, epidemiology, differential diagnosis, pathogenesis, treatment, and prevention, as well as relevant host immune response mechanisms. The session was divided into six main sections (Figure). A detailed description of each component, with suggestions for implementation, can be found in Appendix B. Briefly, the six sections of the session were as follows:

1. A 10-minute pretest consisting of eight multiple-choice questions to determine students' baseline levels of knowledge (Appendix C).
2. A 10-minute didactic lecture reviewing concepts of mucosal immunity of the respiratory tract (Appendix D, slides 1-15).
3. A 35-minute cloud-based portion in which students used a cloud-based spreadsheet (Appendix A) containing several open-ended questions related to *M tuberculosis* while facilitators provided real-time feedback (Appendix E). Appendix F contains a training document with examples of how to provide guided instant feedback.
4. A 35-minute lecture, provided by two faculty members, integrating microbiology and immunology concepts about *M tuberculosis* (Appendix D, slides 17-38) while reviewing and providing class-wide feedback on the material covered in the cloud-based portion above. Appendix D (slides 24-25, 35-36, 40, and 43) contains additional multiple-choice questions that can be used during the class on any real-time student engagement system to promote student learning from the cloud-based portion.
5. A 10-minute posttest measuring learning and helping students with retention of the concepts covered during the session (Appendix C has these questions plus feedback for students).
6. After the session, a review of the student-completed version of Appendix A for accuracy and completion by faculty, who then posted it online for students to use as a study guide.

Data Analysis and Questionnaire

Upon completion of the session, consenting students received access to an optional questionnaire to evaluate their satisfaction during the session and to collect suggestions for improvements in order to better understand how the session might facilitate student learning. The questionnaire gathered basic demographics as well as answers to general satisfaction questions. Questions asked on the questionnaire are listed in Table 1.

The session contained pre- and postformative assessments to determine student progress and incorporated a survey to evaluate student satisfaction and solicit ideas for improvements. The data from the pre- and posttests were analyzed using a paired *t* test with a .001 two-sided significance level.

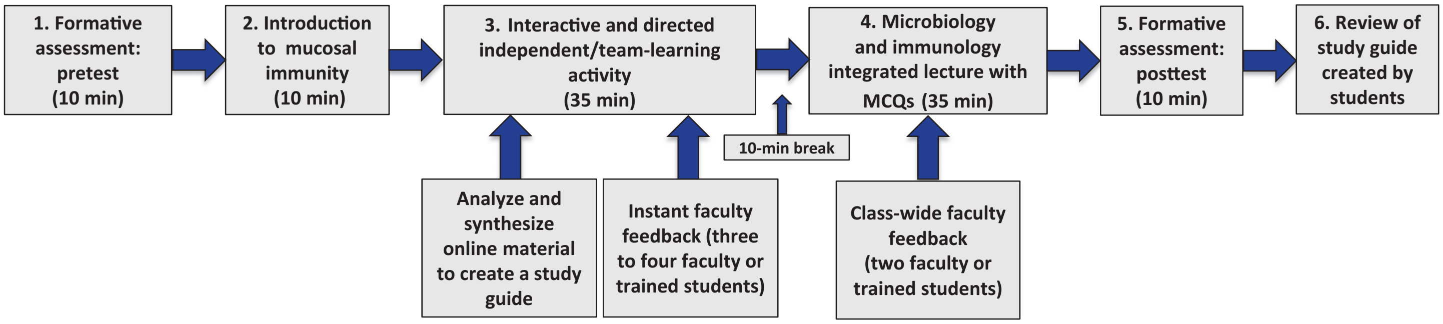

Figure. Schematic of the tuberculosis session: First-year medical students in the respiratory course worked in teams to complete a table using an online collaborative spreadsheet, while faculty provided real-time feedback. The learning management system was used to deliver the pre- and posttests to measure students' learning and performance. After the session, faculty reviewed the student-created document for consistency and accuracy, then posted the study guide in the learning management system. Detailed information about each component of this figure is given in Appendix B. Abbreviation: MCQs, multiple-choice questions.

Copyright © 2021 Jackman et al. This is an open-access publication distributed under the terms of the Creative Commons Attribution license.

Table 1. Student Questionnaire Responses

Theme (*N*)	Likert-Scale Score: *M* (*SD*)	Related Questions in the Questionnaire	Slightly Satisfied/Agree and Strongly Satisfied/Agree:% of Students (No.)
Satisfaction (110)[a]	4.5 (0.9)	How satisfied are you with the lecture portion of CL?	84 (92)
		How satisfied are you with the activity using Google Drive during CL?	69 (76)
		How satisfied are you with the iClicker questions provided during CL?	97 (107)
		How satisfied are you with your experience working as a team during CL?	87 (96)
		Overall, how was your experience with CL including the lecture and activity?	75 (77)
Organization (112)[b]	4.6 (1.1)	The content of CL was well organized.	82 (91)
Knowledge (112)[b]	4.3 (1.0)	CL improved my knowledge about infectious diseases of the lung.	85 (95)
		CL improved my knowledge about mucosal immunity.	81 (91)
Feedback (110)[b]	4.7 (1.1)	During CL, the faculty's feedback was helpful while completing the table.	90 (99)
Preparation time (100)[c]	1.4 (0.8)	On average, how much time do you spend preparing for CL?	70[d] (70)

Abbreviation: CL, cloud-based learning.
[a]Rated on a 6-point Likert scale (1 = *strongly dissatisfied*, 2 = *dissatisfied*, 3 = *slightly dissatisfied*, 4 = *slightly satisfied*, 5 = *satisfied*, 6 = *strongly satisfied*).
[b]Rated on a 6-point Likert scale (1 = *strongly disagree*, 2 = *disagree*, 3 = *slightly disagree*, 4 = *slightly agree*, 5 = *agree*, 6 = *strongly agree*).
[c]Rated on a 5-point Likert scale (1 = *<30 minutes*, 2 = *31-60 minutes*, 3 = *61-90 minutes*, 4 = *91-120 minutes*, 5 = *>120 minutes*).
[d]Percentage of respondents who said they spent less than 30 minutes preparing for the CL session.

This study, including the questionnaire and access to deidentified student pre- and posttest scores, was approved by the Oakland University Institutional Review Board (IRB study number 840485).

Results

To evaluate the session, we used both the student satisfaction questionnaire and pre- and postsession multiple-choice questions. The integrated session was used to deliver immunology and microbiology of *M tuberculosis* over 4 years; the total number of students completing the survey was 114. The median age of participants was 23.8 years ($SD = 1.6$ years). Thirty-seven percent of participants were male, and 63% were female.

Student satisfaction with the session (five questions) was measured on a 6-point Likert scale (1 = *strongly dissatisfied,* 6 = *strongly satisfied*). The mean response was 4.5 ($SD = 0.9$, $n = 110$), indicating that on average, students were slightly satisfied to satisfied with the instructional session. The percentage of students who responded slightly satisfied or strongly satisfied ranged from 69% to 97% (average: 83%). Student perceptions of organization (one question), knowledge (two questions), and feedback received (one question) for the session were measured on a 6-point Likert scale (1 = *strongly disagree,* 6 = *strongly agree*). The mean response for organization was 4.6 ($SD = 1.1$, $n = 112$), for knowledge was 4.3 ($SD = 1.0$, $n = 112$), and for feedback received was 4.7 ($SD = 1.1$, $n = 110$). Student preparation time was measured on a 5-point Likert scale (1 = *<30 minutes,* 5 = *>120 minutes*). The mean response was 1.4 ($SD = 0.8$, $n = 100$), with 70% of students indicating that they needed less than 30 minutes of preparation time. See Table 1 for more details.

Overall, the effectiveness of the session as a teaching tool was measured by comparing mean scores on the pretest to those on the posttest. The average number correct on the pretest was 1.2 out of 6 ($SD = 1.1$, 20%), while the average number correct on the posttest was 3.1 out of 6 ($SD = 1.5$, 51%), indicating that student performance significantly increased 31% ($p < .001$), with a percentage increase on individual questions ranging from 7% to 48%, when comparing the mean of the posttest to the mean of the pretest (Table 2).

Discussion

We developed an interactive TB instructional session integrating microbiology and immunology in the curriculum that allowed medical students to learn these subjects while practicing directed-independent learning using technology. The session required no student preparation, promoted active learning, and facilitated student engagement and collaboration while students created their own study guide.

Students' responses to our survey indicated that they viewed the session as a useful resource for their learning (Table 1), with moderate responses on satisfaction, organization, faculty feedback, and knowledge. Students' perceptions of overall satisfaction, session organization, knowledge gained, and faculty feedback obtained were slightly satisfied to satisfied (Table 1),

Table 2. Pretest and Posttest Results

Score (*N* = 127)	*M* (*SD*, % Correct)	Mean Difference (%)	Standard Error of the Mean	Significance (2-Tailed)
Pretest	1.2 (1.1, 20%)			
Posttest	3.1 (1.5, 51%)	1.9 (31%)	0.1	<.001[a]

[a]Four out of six questions contributed to the significant *p*.

Copyright © 2021 Jackman et al. This is an open-access publication distributed under the terms of the Creative Commons Attribution license.

while students reported very minimal preparation time (between 0 and 30 minutes). We anticipate that more exposure in the curriculum to this new teaching modality may result in higher satisfaction scores.

Based on the pretest and posttest comparison, students did demonstrate learning (Table 2), with an overall increase of 2.5 in the mean (31% points higher on the posttest). While an increase was shown, the average percent correct on the posttest was only 50%. This low average may be due to students working on narrow portions of the study guide or to the difficulty of the posttest questions, suggesting that students need to review the entire class-created study guide to gain a complete knowledge of TB. It is also possible that two questions (questions one and four) were more difficult or that the topic was not sufficiently covered earlier in the curriculum or during the session. To rule out the possibility that this increase was due to memorization of the questions, in some years we included two new questions that students had not seen on either the pretest or the posttest. We found a comparable increase in the mean percent correct when comparing the pretest average score to the posttest average score for those two questions. This suggests that the students were not memorizing but actively learning during the session.

Integration of immunology and microbiology in the curriculum has been done successfully,[21] and integration of disciplines has been shown to facilitate learning in medical education.[22,23] We designed this session to integrate the disciplines of immunology and microbiology without increasing time in the curriculum. To our understanding, there are few publications in *MedEdPORTAL* that integrate immunology with microbiology,[24-27] and none of them describe the immunologic and microbiologic aspects related to TB infection.

To successfully execute this session, there were some challenges. During the session in 2018, there were technical issues with internet bandwidth, which we resolved in class by having two members of each group act as team scribes on the spreadsheet in order to limit individual student internet usage. We also recognize the limitations placed by prior knowledge of cloud-based editing programs, in that faculty or students may have to be trained in how to use the new system. We recommend having four to six students per group, as larger groups increase distraction of students, while smaller groups place increased demand on individuals and decrease the efficiency of the group. Furthermore, at our school, we had the option of presenting the material in two different classrooms. One classroom allowed for presentation of the spreadsheet and the lecture simultaneously on two separate screens, which was preferred as it prevented delays in switching programs. However, when this was unavailable, a single projector screen sufficed. Although it is possible to ask individual teams to complete the entire study guide, doing so would pose a significant burden on each group individually, as well as on time in class, not to mention requiring faculty to spend a large amount of time reviewing each group's work for accuracy while providing feedback. We also recognize some limitations of this cloud-based activity requiring three to five trained faculty or students during the spreadsheet-based group portion, as well as prior knowledge of microbiology and immunology.

There is a need to implement online or remote instructions in medical education, especially when public health measures prevent face-to-face classroom settings. This session could be easily modified for implementation online as a flipped-classroom instruction or for different subjects following the process described in the Figure. Google Drive, which is freely available, allows students to work remotely on the cloud-based portion of the session while instructors provide feedback either in real time or asynchronously. The lecture portion can be presented live or recorded, offering feedback to the entire class or in groups (as described in Appendix B, section 3) using any available streaming software (e.g., Panopto, Zoom, WebEx). Although Google Drive was used in our study, other alternative collaboration software (e.g., Microsoft OneDrive) can be used to work on the student spreadsheet. Investigations to evaluate the effectiveness of this session online are currently underway.

We have described a novel instructional session about *M tuberculosis* that integrates immunology and microbiology and effectively increases student knowledge without increasing advance preparation time. In addition, this instruction applies constructivist pedagogy[28] and utilizes TPACK[19] and generative learning[20] as students analyze and synthesize information in order to create their own study guide while receiving immediate feedback on their work from faculty. Future analysis to determine whether this session improves the learning outcomes of medical students, as well as its effectiveness on long-term retention and learning when compared with other instructional sessions, is warranted.

Appendices

A. Student Mycobacterial Defense Mechanisms Spreadsheet.xlsx
B. Cloud-Based Learning Detailed Description.docx
C. Pretest and Posttest MCQs.docx

Copyright © 2021 Jackman et al. This is an open-access publication distributed under the terms of the Creative Commons Attribution license.

D. Mycobacterium tuberculosis Defense Mechanisms.pptx

E. Instructor Mycobacterial Defense Mechanisms Spreadsheet.xlsx

F. Feedback Guidelines for Cloud-Based Learning.docx

All appendices are peer reviewed as integral parts of the Original Publication.

Trent D. Jackman: Fourth-Year Medical Student, Oakland University William Beaumont School of Medicine

Anne M. Dersch, MD: First-Year Ophthalmology Resident, University of Arizona College of Medicine

Tracey A. H. Taylor, PhD: Associate Professor, Department of Foundational Medical Studies, Oakland University William Beaumont School of Medicine; ORCID: https://orcid.org/0000-0002-4739-7127

Claudio Cortes, DVM, PhD: Assistant Professor, Department of Foundational Medical Studies, Oakland University William Beaumont School of Medicine; ORCID: https://orcid.org/0000-0003-2870-0028

Acknowledgments
Trent D. Jackman and Claudio Cortes contributed equally to this work. We thank Inaya Hajj Hussein, PhD, Mary Dereski, PhD, Stefan Walter, PhD, Paul Megee, PhD, and Dwayne Baxa, PhD, for providing student feedback during the sessions, and Daniel Gildner, MS, for statistical analysis and support.

Disclosures
None to report.

Funding/Support
None to report.

Ethical Approval
The Oakland University Institutional Review Board approved this study.

References

1. Dowdle WR; Centers for Disease Control. A strategic plan for the elimination of tuberculosis in the United States. *MMWR Suppl*. 1989;38(3):1-25.
2. Reported tuberculosis in the United States, 2018. Centers for Disease Control and Prevention. Updated September 6, 2019. https://www.cdc.gov/tb/statistics/reports/2018/default.htm
3. Miramontes R, Hill AN, Woodruff RSY, et al. Tuberculosis infection in the United States: prevalence estimates from the National Health and Nutrition Examination Survey, 2011-2012. *PLoS One*. 2015;10(11):e0140881. https://doi.org/10.1371/journal.pone.0140881
4. *Global Tuberculosis Report 2019*. World Health Organization; 2019. https://apps.who.int/iris/bitstream/handle/10665/329368/9789241565714-eng.pdf
5. Furin J, Cox H, Pai M. Tuberculosis. *Lancet*. 2019;393(10181):1642-1656. https://doi.org/10.1016/S0140-6736(19)30308-3
6. Reid MJA, Arinaminpathy N, Bloom A, et al. Building a tuberculosis-free world: the *Lancet* Commission on tuberculosis. *Lancet*. 2019;393(10178):1331-1384. https://doi.org/10.1016/S0140-6736(19)30024-8
7. Ou Y, Luo Z, Mou J, et al. Knowledge and determinants regarding tuberculosis among medical students in Hunan, China: a cross-sectional study. *BMC Public Health*. 2018;18:730. https://doi.org/10.1186/s12889-018-5636-x
8. Drain PK, Primack A, Hunt DD, Fawzi WW, Holmes KK, Gardner P. Global health in medical education: a call for more training and opportunities. *Acad Med*. 2007;82(3):226-230. https://doi.org/10.1097/ACM.0b013e3180305cf9
9. Hau DK, Smart LR, DiPace JI, Peck RN. Global health training among U.S. residency specialties: a systematic literature review. *Med Educ Online*. 2017;22(1):1270020. https://doi.org/10.1080/10872981.2016.1270020
10. Aaron LS, Roche CM. Teaching, learning, and collaborating in the cloud: applications of cloud computing for educators in post-secondary institutions. *J Educ Technol Syst*. 2011;40(2):95-111. https://doi.org/10.2190/ET.40.2.b
11. Wood M. Collaborative lab reports with Google Docs. *Phys Teach*. 2011;49(3):158-159. https://doi.org/10.1119/1.3555501
12. Marra RM, Steege L, Tsai CL, Tang NE. Beyond "group work": an integrated approach to support collaboration in engineering education. *Int J STEM Educ*. 2016;3:17. https://doi.org/10.1186/s40594-016-0050-3
13. Orndorff HN III. Collaborative note-taking: the impact of cloud computing on classroom performance. *Int J Teach Learn High Educ*. 2015;27(3):340-351.
14. Premkumar K, Pahwa P, Banerjee A, Baptiste K, Bhatt H, Lim HJ. Does medical training promote or deter self-directed learning? A longitudinal mixed-methods study. *Acad Med*. 2013;88(11):1754-1764. https://doi.org/10.1097/ACM.0b013e3182a9262d
15. Quinones J. A simulated patient encounter of status epilepticus. *MedEdPORTAL*. 2008;4:772. https://doi.org/10.15766/mep_2374-8265.772
16. Morehead R, Fons R. Diagnosis Quest. *MedEdPORTAL*. 2010;6:8134. https://doi.org/10.15766/mep_2374-8265.8134
17. Lee P, Johnson A, Rajashekara S, et al. Clinical topics in global health: a practical introduction for pre-clinical medical students. *MedEdPORTAL*. 2013;9:9471. https://doi.org/10.15766/mep_2374-8265.9471
18. Tegzes J, Mackintosh S, Meyer T, Redman-Bentley D, Aston S. To be or not to be: an interprofessional problem-based learning

Copyright © 2021 Jackman et al. This is an open-access publication distributed under the terms of the Creative Commons Attribution license.

case introducing the One Health Initiative. *MedEdPORTAL*. 2013;9:9623. https://doi.org/10.15766/mep_2374-8265.9623

19. Mishra P, Koehler MJ. Technological pedagogical content knowledge: a framework for teacher knowledge. *Teach Coll Rec*. 2006;108(6):1017-1054.
20. Wittrock MC. Learning as a generative process. *Educ Psychol*. 2010;45(1):40-45. https://doi.org/10.1080/00461520903433554
21. Willey JM, Lim YS, Kwiatkowski T. Modeling integration: co-teaching basic and clinical sciences medicine in the classroom. *Adv Med Educ Pract*. 2018;9:739-751. https://doi.org/10.2147/AMEP.S169740
22. Irby DM, Cooke M, O'Brien BC. Calls for reform of medical education by the Carnegie Foundation for the Advancement of Teaching: 1910 and 2010. *Acad Med*. 2010;85(2):220-227. https://doi.org/10.1097/ACM.0b013e3181c88449
23. Goldman E, Schroth WS. Deconstructing integration: a framework for the rational application of integration as a guiding curricular strategy. *Acad Med*. 2012;87(6):729-734. https://doi.org/10.1097/ACM.0b013e318253cad4
24. Manglik N, Dudrey EF, Baatar D, Piskurich JF. Immune response to bacteria: an integrated learning module to enhance preclinical students' competency in immunology. *MedEdPORTAL*. 2017;13:10585. https://doi.org/10.15766/mep_2374-8265.10585
25. Dudrey EF, Manglik N, Velasco VY, Baatar D, Piskurich JF. Innate and adaptive immune response to a virus: an integrated learning module. *MedEdPORTAL*. 2018;14:10757. https://doi.org/10.15766/mep_2374-8265.10757
26. Wald D, Buttaro B. A workshop combining simulation and self-directed learning to teach medical students about pneumonia. *MedEdPORTAL*. 2016;12:10390. https://doi.org/10.15766/mep_2374-8265.10390
27. Bramblett D, McMahon K, Piskurich J, Simpson G. Infective endocarditis TBL. *MedEdPORTAL*. 2011;7:8287. https://doi.org/10.15766/mep_2374-8265.8287
28. Richardson V. Constructivist pedagogy. *Teach Coll Rec*. 2003;105(9):1623-1640.

Received: June 5, 2020
Accepted: February 11, 2021
Published: April 14, 2021

Copyright © 2021 Jackman et al. This is an open-access publication distributed under the terms of the Creative Commons Attribution license.
